# Central veno-arterial ECMO via an aortic prosthetic graft as significant alternative to peripheral ECMO cannulation: evaluation of patient outcomes in infarct-related cardiogenic shock

**DOI:** 10.1186/s13019-026-03849-9

**Published:** 2026-02-04

**Authors:** Boris Kuzmin, Juliana Ponomarenko-Jakunin, Mohammad Fadel, Tigran Gross, Sam Varghese, Olaf Keyser, Fridtjof Scholz, George Awad, Alexander Schmeisser, Jens Wippermann

**Affiliations:** 1 Department of Cardiothoracic Surgery, University Hospital, Leipziger str. 44, Magdeburg, 39120 Germany; 2 Department of Cardiology and Angiology, University Hospital, Magdeburg, Germany

**Keywords:** Cardiogenic shock, Acute myocardial ischemia, Mechanical circulatory support, Extracorporeal membrane oxygenation, Hemothorax

## Abstract

**Background:**

For patients presenting with cardiogenic shock due to acute myocardial ischaemia, veno-arterial extracorporeal membrane oxygenation (ECMO) is often the only potential lifesaving intervention remaining when revascularisation does not immediately improve the patient’s condition. A relatively recent cannulation technique involves using an end-to-side prosthetic graft sewn onto the ascending aorta to allow the inflow cannula to be inserted outside the chest. There is no literature on this cannulation method compared to traditional peripheral ECMO. We performed a comprehensive analysis to evaluate the advantages and disadvantages of this technique providing valuable insights into patient outcomes.

**Methods:**

A total of 65 patients suffering from severe, infarct-related cardiogenic shock were supported with ECMO at the University Hospital Magdeburg between 2014 and 2022. The patients were divided into two groups: a central ECMO group of 33 patients and a peripheral ECMO group consisting of 32 patients. We compared the pre-implantation laboratory parameters as well as laboratory parameters on the third day of ECMO, post-implanatation complications, and survival rates in both the groups.

**Results:**

The primary outcome of the study was survival. Patients who received central ECMO exhibited a higher 60-day survival rate (54.5%) in comparison to those who received peripheral ECMO (28.1%) (*p* = 0.031). With regard to secondary outcomes, patients receiving central ECMO exhibited a higher incidence of bleeding events (56.3% versus 84.8%, *p* = 0.011), along with a greater prevalence of hemothorax (21.9% versus 53.1%, *p* = 0.013) and mucosal bleeding (12.5% versus 48.5%, *p* = 0.002). Conversely, patients with peripheral ECMO exhibited a higher incidence of acute leg ischaemia (18.8% vs. 3.0%, *p* = 0.041).

**Conclusion:**

The application of central ECMO with a prosthesis on the ascending aorta can effectively support patients experiencing cardiogenic shock due to acute myocardial ischaemia. However, more careful hemostasis is required to minimise bleeding, especially hemothorax.

## Introduction

 Acute myocardial infarction (AMI) results in cardiogenic shock (CS) in 5–8% of the pateints [1, 2]. According to the European Society of Cardiology guidelines, patients in CS experience a decrease in systolic blood pressure < 90 mm Hg despite appropriate fluid resuscitation. The onset of clinical and laboratory evidence of end-organ failure is followed by cold extremities, oliguria, narrow pulse pressure, metabolic acidosis, elevated serum lactate, and elevated serum creatinine [3]. Initial therapy for cardiogenic shock includes infusion therapy, vasopressors and inotropes, which, according to the 2017 guidelines, can be considered to stabilize hemodynamics [4]. The most important management of cardiogenic shock in AMI remains the urgent restoration of blood flow in the culprit coronary artery. Even after successful revascularisation, many patients continue to experience refractory CS. In such cases, veno-arterial extracorporeal membrane oxygenation (ECMO) can be used to provide mechanical circulatory support (MCS) to patients with acute left or biventricular failure, when neither medical therapy nor revascularisation leads to hemodynamic stabilisation. Three principal techniques for ECMO cannulation have been described in the literature: (1) peripheral cannulation (pECMO), which is performed by puncturing the femoral artery and vein; (2) an alternative peripheral configuration, employing the conventional venous access route, via either the femoral or internal jugular vein, with arterial return to a graft positioned on the subclavian artery; (3) central ECMO (cECMO), involving cannulation of the aorta and right atrium (or both venae cavae), often leaving the cannula from cardiopulmonary bypass in position if weaning is not possible due to CS after bypass surgery [5–7]. Most publications on cECMO support focus on patients with postcardiotomy cardiogenic shock, which means that these patients are heterogeneous in terms of disease (cardiogenic shock due to AMI, severe heart reoperation, endocarditis, myocarditis, etc.).

Central cannulation provides adequate venous drainage and more effective cardiac decompression than peripheral cannulation [6]. In addition, since oxygenated blood is returned to the ascending aorta physiological antegrade blood flow and upper body hypoxemia are avoided in comparison to pECMO. The most significant drawbacks of central ECMO, where cannulas are inserted directly into the aorta and right atrium, are the requirement for chest reentry, thereby increasing the risk of bleeding, the potential for repeated surgical procedures, and the possibility of developing mediastinitis [6]. The main advantage of peripheral cannulation is that it is easy to perform using the Seldinger technique and does not require surgery, often being carried out at the patient’s bedside under sterile conditions. Disadvantages include upper body hypoxemia, thrombus formation in the aortic root, left ventricular distension, and risk of lower limb ischaemia [5, 6, 8]. Since both methods of cannulation are still associated with significant limitations, new methods of cannulation that maintain the benefits and eliminate the complications of MCS should be explored.

The technique of a prosthesis being anastomosed to the aorta or a major vessel for temporary use to connect an arterial ECMO cannula has been described previously, but only in a small group of patients as case reports [9–11].

In our study, conducted on a cohort of 65 patients, we compare the results of relatively novel cannulation method. The method involves the extraction of venous blood through the femoral vein, with oxygenated blood subsequently returned via a prosthetic graft to the ascending aorta.

## Patients and methods

### Study design

**Fig. 1:**
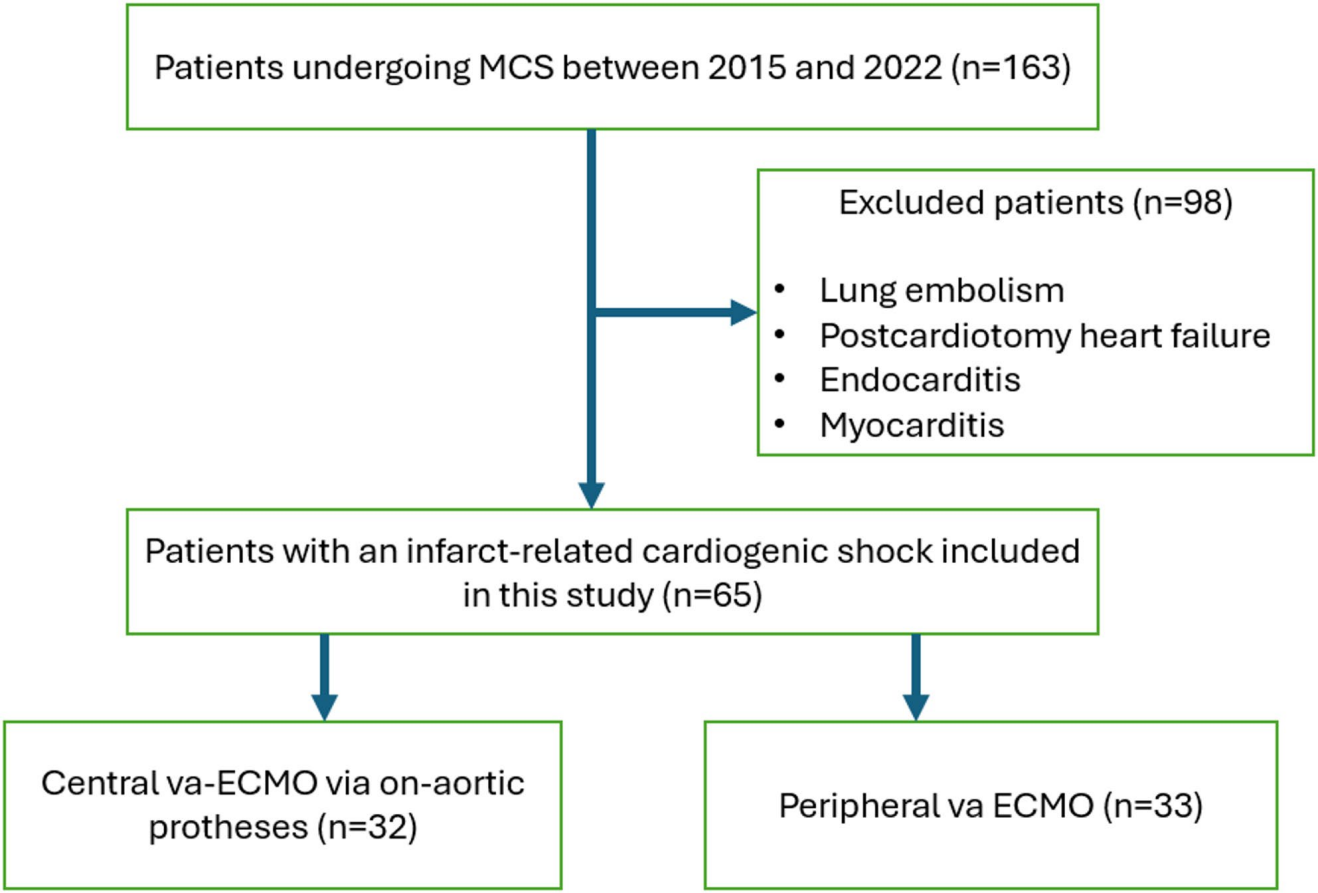
The flow chart of the patients entered the study. MCS – mechanical circulatory support, va-ECMO – veno-arterial extracorporeal membrane oxygenation

This retrospective cohort study used databases of intensive care unit (ICU) admissions from 2014 to 2022 at University Hospital Magdeburg. The MCS was established in the event of infarct-related CS following unsuccessful weaning from cardiopulmonary bypass in the operation theater or postoperative refractory CS in the ICU in patients who have received revascularisation in the form of a percutaneous coronary intervention (PCI) or coronary artery bypass graft (CABG). We excluded patients with CS due to prolonged reoperations or complications from elective cardiac surgery requiring ECMO (postcardiotomy heart failure), as well as patients with endocarditis, pulmonary embolism or severe myocarditis. The present study focuses on patients suffering from CS following a myocardial infarction. This approach ensured that the group of patients was homogeneous, with all suffering from a single disease (Fig. 1).

The baseline covariates consisted of the following variables: demographics (age and gender), comorbidities, laboratory parameters, operation duration, and therapy approach. The clinical endpoints included survival, length of hospital stay, length of stay in the ICU, duration of mechanical ventilation, and postoperative complications. Time-varying covariates included laboratory parameters before and during MCS, as well as any complications observed during this period.

### ECMO settings

The clinic utilises three different ECMO systems: CardioHelp, Rotaflow II, and Rotaflow I (Getinge AB, Rastatt, Germany). Additionally, HLS cannulas and PLS sets (Getinge AB, Rastatt, Germany) were used. To prevent thromboembolic complications, an anticoagulation protocol involving the intravenous infusion of unfractionated heparin was implemented, with a target activated clotting time (ACT) values ranging from 180 to 200 s and activated partial thromboplastin time (aPTT) values between 50 and 60 s. ECMO parameters varied among patients, with blood flow ranging from 3.0 to 4.9 l per minute, gas flow from 2 to 5 l per minute, and rotational speed from 3,000 to 5,000 rotations per minute. The fraction of inspired oxygen (FiO₂) on the ECMO device was initially maintained at 80–100% and decreased as the patient’s condition had improved.

### Cannulation methods

**Fig. 2:**
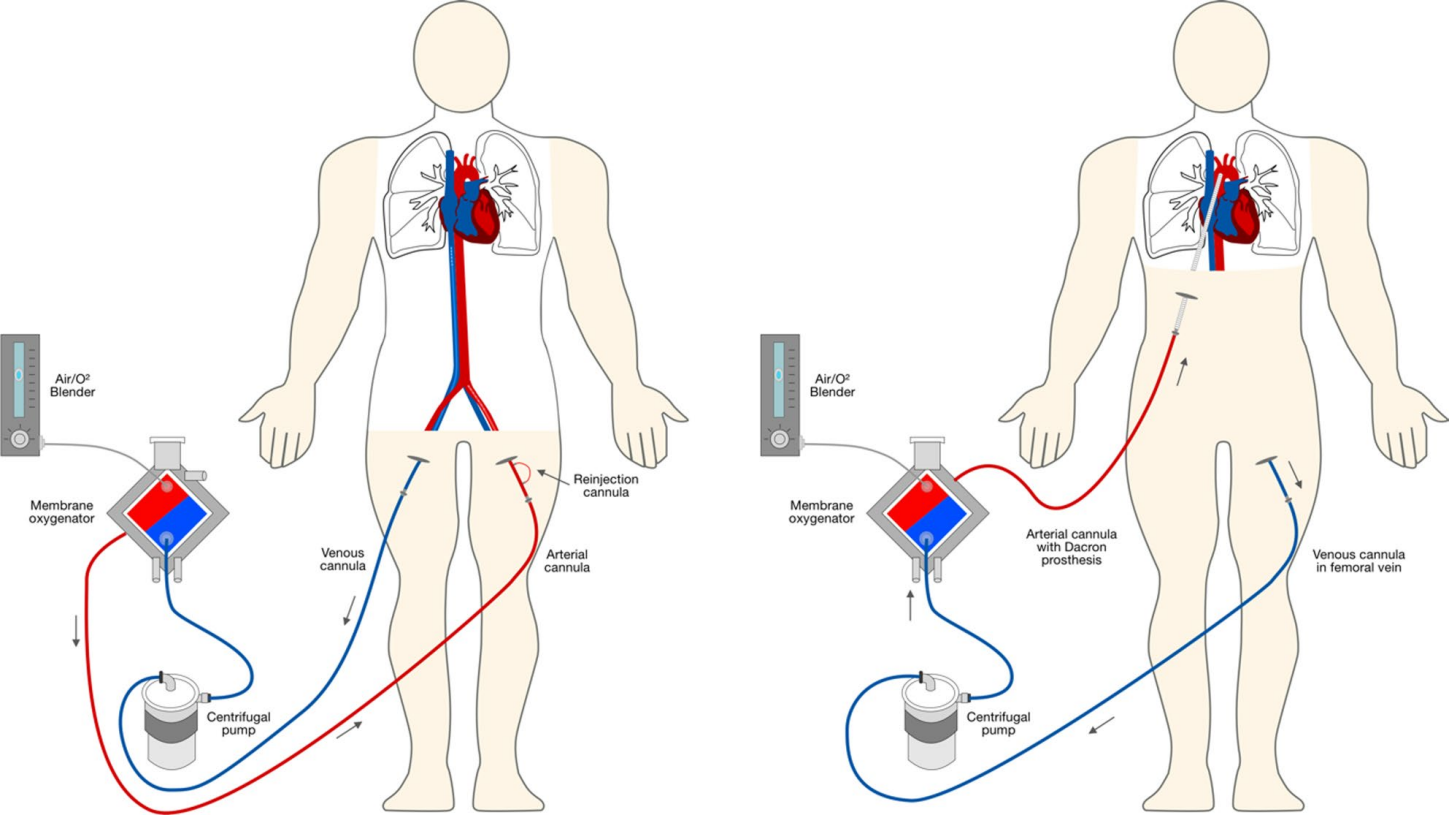
Peripheral veno-arterial ECMO (left) and central veno-arterial ECMO via an on-aortic prosthetic graft

Peripheral cannulation (Fig. 2) involves draining venous blood from the inferior vena cava via a cannula (21–23 Fr) inserted into the femoral vein. Typically, the arterial cannula is a short 17–21 Fr catheter inserted into the femoral artery up to the common iliac artery. To avoid leg ischemia, all patients in the pECMO group received an 8 Fr distal antegrade cannula placed in the femoral artery and connected to an ECMO circuit to ensure sufficient leg perfusion. The cannulation was performed percutaneously and with ultrasound guidance.

We define cECMO as central as the oxygenated blood is returned to the ascending aorta resulting in antegrade blood supply. In our patients, we used an eight-millimetre polytetrafluoroethylene prosthesis, which has demonstrated excellent results in vascular surgery in the recent years [12]. The prosthesis was sutured to the ascending aorta using the “end-to-side” anastomosis technique. It was then tunneled through the chest and positioned below the rib cage on the anterior abdominal wall. Outside the body cavity, the prosthetic graft is connected to the ECMO cannula and the circuit. Venous cannulation is typically performed through the femoral vein using the Seldinger technique with the cannula pushed till the right atrium, which is guided intraoperatively by transoesophageal echocardiography. With this cannulation method, the chest can be closed completely after the first procedure. To remove the arterial cannula in case of a successful ECMO weaning could be done on bedside by detaching the cannula from the prosthetic graft, which is secured with ligatures and plunged under the subcutaneous tissue.

The procedure of central cannulation with a similar method using a prosthetic graft was previously described in the literature as Y-ECMO [11]. The key difference between this procedure and others is that the left and right atria are cannulated directly and connected by a Y-connector to the ECMO circuit for inflow.

### Postoperative management

In the early postoperative period following the application of ECMO, we aim to reduce catecholamine support (dobutamine, epinephrine), unless the patient exhibits signs of unfavourable right ventricular failure. In cases of right ventricular failure support was given though iloprost inhalation therapy, enoximone therapy and nitric oxide respiratory ventilation were administered. The main objective during the subsequent 7–10 days after ECMO initiation is to wean the patient off ECMO while monitoring them with control echocardiograms. If weaning is not possible, a decision is made on an individual basis as to whether the patient is a suitable candidate for a left ventricular assist device (LVAD). This decision takes into account current complications, including neurological status, as well as the patient’s age and social circumstances.

### Outcomes

The primary endpoint was the survival rate after 60 days of ECMO initiation. The secondary endpoints encompassed complications such as major bleeding events (e.g. cerebral, gastrointestinal, hemothorax, cannulation-site bleeding, pericardial and retroperitoneal bleeding), organ failures (acute kidney injury, liver failure and lung failure), thrombosis, the duration of MCS and laboratory parameters. These values and incidences were examined in the central versus peripheral ECMO analysis.

### Statistical analysis

Baseline patient demographics, including data on age, sex, body mass index (BMI) and comorbidities, were compared between the two groups alongside outcomes, duration of MCS, laboratory parameters, and complications such as bleeding events, stroke, acute kidney injury, acute liver failure, pneumothorax, pulmonary embolism, acute abdomen, thrombocytopenia, and infections. Statistical analysis was performed using the SPSS software package (version 29, IBM, Armonk, New York, USA). The normality of the data was evaluated through the application of the Kolmogorov-Smirnov test. In order to facilitate univariate comparisons of numerical variables, the two-tailed, independent t-test and Mann-Whitney U test were employed. Categorical variables were analysed using the chi-squared or Fisher’s exact test and presented as number (percentage). Numerical variables with a normal distribution are presented as mean ± standard deviation, while those without a normal distribution are presented as median (interquartile range; IQR). The significance level was set at α = 0.05. The Kaplan-Meier method was employed to estimate the survival curve. Multivariate logistic regression was used to identify the factors associated with 60-day mortality in the entire cohort. Variables observed both before and during ECMO, as well as the complications, were processed using the logistic regression method [8]. Variables demonstrating a p value of less than 0.05 in the logistic regression process were then re-tested using the logistic regression method with an inclusion selection procedure to construct the final prediction model. Graphs were created using Microsoft Excel^®^ for Windows 10.

## Results

### Baseline characteristics

Among the total of 163 patients who received perioperative va-ECMO during the study period, 98 patients were excluded from the study. Ultimately, 65 patients with infarct-related CS were enrolled in this study. These patients had previously undergone revascularisation via PCI, or aortocoronary bypass surgery in cases where PCI was not possible due to the complexity of coronary artery stenosis. The patients were divided into two groups: central va-ECMO (cECMO group) versus peripheral va-ECMO (pECMO group). The MCS duration of the central and peripheral va-ECMO groups was 130.0 (61.5–178.0) and 99.5 (34.0–163.3.0.3) hours, respectively (*p* = 0.198). Firstly, the difference between the groups is that the patients who received pECMO were younger, with a mean age of 62.0 ± 10.2 years (65.0 ± 9.7 years in the cECMO group; 59.0 ± 9.9 years in the pECMO group, *p* = 0.016). Among the study population, 42 patients (64.6%) were male and 23 (35.4%) were female. There was no significant difference in comorbidities between the two groups. Secondly, patients in the pECMO cohort exhibited a higher propensity for undergoing PCI (2 [6.1%] vs. 13 [40.6%], *p* = 0.001). This finding is reasonable, as opening of the chest to implant cECMO was avoided in cases of CS after successfull treatment of culprit leisions with PCI, thereby pECMO was preferred by these patients. Two patients who underwent revascularisation via PCI received cECMO due to cardiogenic shock accompanied by left ventricular rupture, which required left ventricular repair. Accordingly, CABG was performed more frequently in the cECMO group (24 [72.7%] vs. 14 [43.8%], *p* = 0.017), while the number of combined interventions of CABG with a valve surgery was equal between the two groups.

The mean ejection fraction in all patients with cardiogenic shock before intervention in this study was 21.1%± 10.5%. Twelve (18.5%) patients manifested moderate or severe mitral insufficiency. The most prevalent underlying cause of cardiogenic shock was identified as severe lesions affecting two coronary arteries, accounting for 47.7% of the cases. The second most prevalent cause was found to be left anterior descending artery (LAD) lesions, accounting for 18.5% of the cases, followed by left main lesions, which accounted for 16.9% of the cases. The clinical and demographic characteristics of the two groups are summarised in Table 1.

**Table 1:** Demographic and clinical characteristics for the entire population and the cECMO and pECMO groups

Baseline characteristics	Overall, *n* = 65	cECMO group, *n* = 33	pECMO group, *n* = 32	*p*-value
*Preoperative patients chrachtersitics*				
Age	62.0 ± 10.2	65.0 ± 9.7	59.0 ± 9.9	0.016
Male	42 (64.6%)	18 (54.5%)	24 (75.0%)	0.085
BMI	27.5 ± 4.6	26.4 ± 3.1	28.6 ± 5.5	0.060
Time of admission before ECMO (h)	15.0 (7.0–61.5.0.5)	19.0 (8.0–67.5.0.5)	10.0 (5.0–57.5.0.5)	0.287
Chronic kidney disease	6 (9.2%)	3 (9.1%)	3 (9.3%)	0.968
Hypertension	51 (78,5%)	27 (81.8%)	24 (70.6%)	0.504
Diabetis	18 (27.7%)	10 (30.3%)	8 (25.0%)	0.633
Chronic lung disease	4 (6.2%)	3 (9.1%)	1 (3.1%)	0.317
Smoking	23 (35.4%)	12 (36.4%)	11 (34.4%)	0.867
Peripheral artery disease	6 (9.2%)	5 (15.2%)	1 (3.1%)	0.094
LVEF	21.1 ± 10.5	22.9 ± 10.3	19.2 ± 10.5	0.154
Mitral regurgitation II-III°	12 (18,5%)	8 (24.4%)	4 (11.8%)	0.223
Aortic regurgitation II-III°	6 (9.2%)	4 (12.1%)	2 (6.3%)	0.414
Aortic stenosis II-III°	4 (6.2%)	3 (9.1%)	1 (3.1)	0.317
*Intraoperative/intraprocedural data*				
Infarct caused artery: LMA LAD RCA RCX 2 Vessels	10 (15.4%) 12 (18.5%) 9 (13.8%) 2 (3.1%) 31 (47.7%)	4 (12.1%) 8 (24.4%) 3 (9.1%) 2 (6.1%) 16 (48.5%)	6 (18.8%) 4 (11.8%) 6 (18.8%) 0 (0%) 15 (46.9%)	0.459 0.223 0.259 0.157 0.897
PCI	15 (23.1%)	2 (6.1%)	13 (40.6%)	0.001
CABG	38 (58.5%)	24 (72.7%)	14 (43.8%)	0.017
CABG + Valve operation	12 (18.5%)	7 (21.2%)	5 (15.6%)	0.561
Bypass time (min)	130.5 (97–200)	121.0 (97.0–160.0.0.0)	157.0 (97.0–223.0.0.0)	0.129
Aortic clamp time (min)	56.0 (42–80.0)	54.0 (41.0–72.0)	63.0 (47.0–101.0.0.0)	0.082
Reperfusion (min)	52.5 (34.0–70.8.0.8)	55.5 (33.8–70.80	50.0 (34.3–102.8.3.8)	0.729
*Preoperative medication*				
Clopidogrel/Ticagrelor	24 (36.9%)	11 (33.3%)	13 (40.6%)	0.543
Aspirin	33 (50,8%)	17 (51.5%)	16 (50.0%)	0.902
DOAC/Marcoumar	4 (6,2%)	1 (3.0%)	3 (9.8%)	0.387

ECMO, extracorporeal membrane oxygenation; BMI, body mass index; LVEF, left ventricular ejection fraction; LMA, left main artery; LAD, left anterior descending artery; RCA, right coronary artery; RCX, ramus circumflex artery; PCI, percutaneous coronary intervention; CABG, coronary artery bypass graft; DOAC, direct oral anticoagulants

No significant differences in laboratory values were observed immediately before operative intervention and ECMO, with the exception of worse lactate acidosis (3.0 [2.0–5.5.0.5] vs. 7.4 [4.4–11.0] mmol/l, *p* = 0.001) and correspondingly lower pH (7.33 ± 0.09 vs. 7.26 ± 0.15, *p* = 0.028) in the pECMO group (Table 2).

**Table 2:** Laboratory parameters before MCS application of the entire population and for the cECMO and pECMO groups

Parameters	Overall, *n* = 65	cECMO group, *n* = 33	pECMO group, *n* = 32	*p*-value
pH	7.30 ± 0.13	7.33 ± 0.09	7.26 ± 0.15	0.028
Lactate (mmol/l)	4.8 (2.4–8.3)	3.0 (2.0–5.5.0.5)	7.4 (4.4–11.0)	0.001
Hemoglobin (mmol/l)	7.7 ± 1.5	7.7 ± 1.4	7.6 ± 1.6	0.965
Leucocyte (10^9^/L)	18 ± 6.1	17.5 ± 6.4	18.6 ± 5.8	0.478
Platelet count (10^9^/L)	241.7 ± 94.1	236.2 ± 90.5	246.8 ± 98.6	0.663
CRP (mg/L)	40.2 (7.4–104.5.4.5)	41.7 (7.5–129.0)	37.7 (7.1–97.2)	0.641
CK (µmol/(s•L)	13.8 (4.6–32.8)	17.4 (5.4–41.3)	8.9 (4.3–28.9)	0.804
CK-MB (µmol/(s•L)	2.0 (0.8–5.5)	3.1 (1.0–6.5.0.5)	2.0 (0.7–4.8)	0.438
CK-MB (%)	10.8 (6.9–13.2)	10.8 (7.6–13.2)	11.0 (5.4–13.4)	0.911
PTT (s)	39.1 (29.6–62.8)	40.3 (29.8–51.6)	37.6 (29.0–82.5.0.5)	0.922
INR	1.2 (1.1–1.5)	1.2 (1.1–1.4)	1.3 (1.1–1.5)	0.151
Antithrombin (%)	73.9 ± 19.7	77.2 ± 16.6	70.1 ± 22.4	0.177
Fibrinogen (g/L)	3.9 ± 1.6	4.1 ± 1.2	3.6 ± 1.9	0.227
LDH (µmol/ls)	13.7 (7.9–28.3)	13.2 (8.2–29.3)	13.9 (7.0–27.0)	0.807
ALT (µmol/ls)	1.9 (0.7–6.8)	1.5 (0.6–5.0)	2.9 (0.9–9.3)	0.154
Creatinine (µmol/l)	115.0 (86.5–158.5.5.5)	115.5 (85.5–144.5.5.5)	115.0 (86.5–169.0)	0.542
Total bilirubin (µmol/l)	9.7 (7.1–17.8)	9.7 (7.1–16.0)	9.7 (6.8–18.1)	0.844

### Primary and secondary endpoints

**Fig. 3:**
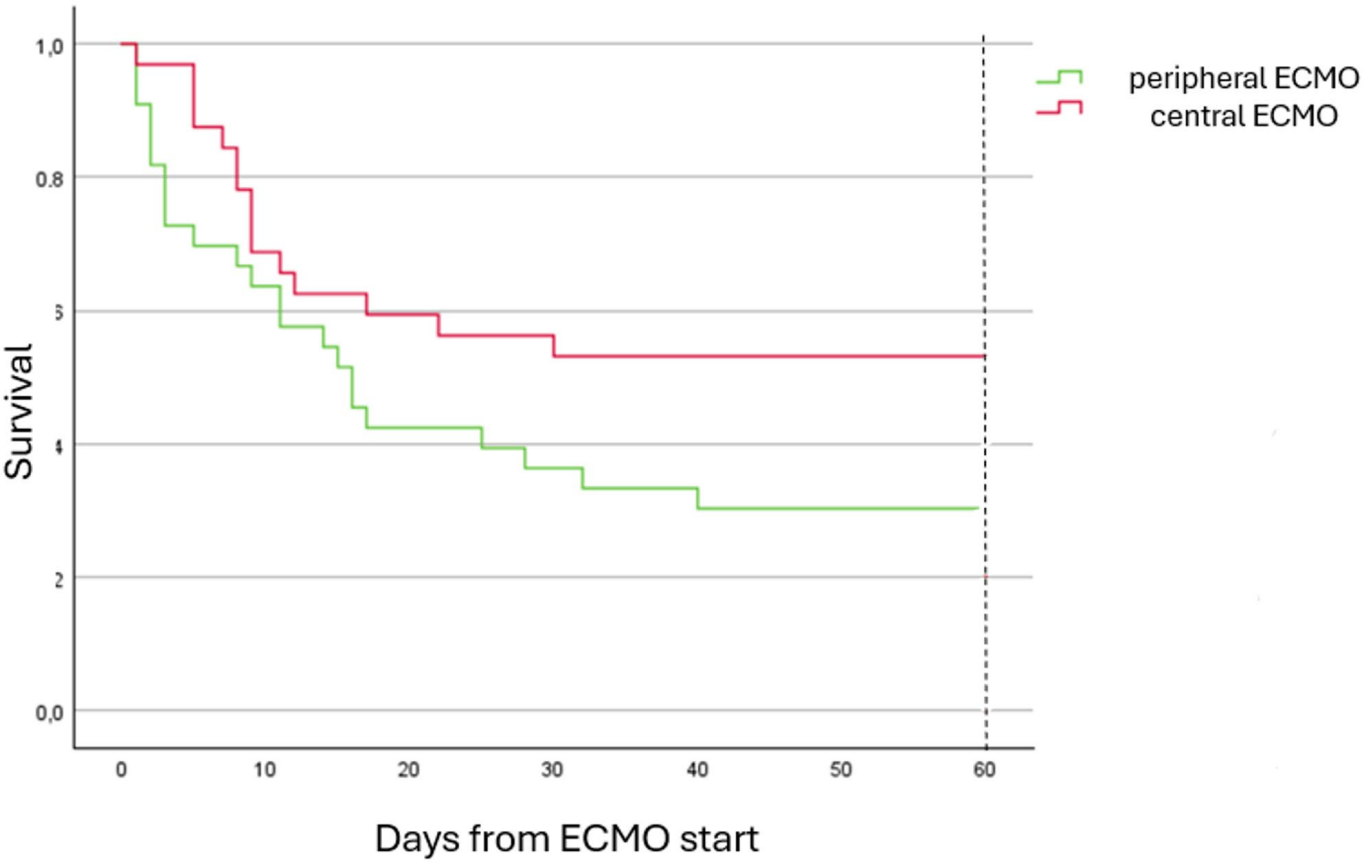
Kaplan-Meier curves for pECMO and cECMO patients. ECMO, extracorporeal membrane oxygenation

The overall 60-day survival for the entire cohort was 41.5% (*n* = 27) (Table 3). Survival rates differed significantly between the two groups, with 54.5% of cECMO patients and 28.1% of pECMO patients surviving 60 days (*p* = 0.031) (Fig. 3). LVAD was implanted in 4 patients, with two patients in each cohort.

**Table 3.** Survival, complications, and characteristics of the therapy during MCS of the entire population and for the cECMO and pECMO groups

Characteristics	Overall, n = 65	cECMO group, n = 33	pECMO group, n = 32	p-value
Survival 60 days	27 (41.5%)	18 (54.5%)	9 (28.1%)	0.031
Duration ECMO (hours)	113.0 (49.5-167.0)	130.0 (61.5-178.0)	99.5 (34.0-163.3)	0.198
ICU LOS all patients (days)	15.0 (8.0-25.0)	17.0 (8.5-32.5)	14.5 (3.5-24.8)	0.253
Total Hospital LOS all patients (days)	16.00 (9.0-26.0)	17.0 (9.0-32.5)	16.0 (2.5-25.0)	0.243
Intubation time (days)	11.0 (4.5-15.0)	11.0 (5.5-14.0)	9.0 (3.0-16.5)	0.969
Circuit Change of ECMO	10 (15.4%)	6 (18.2%)	4 (12,5%)	0.526
Major bleeding events	46 (70,8%)	28 (84.8%)	18 (56.3%)	0.011
- Cerebral	1 (1,5%)	0 (0%)	1 (3.0%)	0.306
- Gastrointestinal	2 (3,1%)	2 (6.1%)	0 (0%)	0.157
- Hemothorax	24 (36,9%)	17 (53.1%)	7 (21.9%)	0.013
- Pericardial tamponade	14 (21,5%)	8 (24.2%)	6 (18.8%)	0.590
- Cannulation side	16 (24.6%)	9 (27.3%)	7 (21.9%)	0.614
- Mucosal/epistaxis	20 (30,8%)	16 (48.5%)	4 (12.5%)	0.002
- Endobronchial	2 (3,1%)	1 (3.0%)	1 (3.0%)	0.982
- Neck-bleeding (tracheostoma)	1 (1,5%)	1 (3.0%)	0 (0%)	0.321
- Surgical wound on the lower leg after fascia splitting due to compartment syndrome	3 (4.6%)	1 (3.0%)	2 (6,3%)	0.536
Re-thoracotomy/patient	0 (0-1.5)	1.0 (0-1.5)	0 (0-0.8)	0.001
Other complications	63 (96,2%)	31 (93,9%)	32 (100%)	0.167
- Lung embolism	3 (4,6%)	0 (0%)	3 (9,8%)	0.072
- Stroke	5 (12,3%)	2 (6.1%)	3 (9,8%)	0.616
- Bacteremia	6 (9,2%)	2 (6,1%)	4 (12,5%)	0.370
- Leg ischemia	7 (10,8%)	1 (3,0%)	6 (18,8%)	0.041
- Renal failure requiring dialysis during ECMO	34 (52,3%)	19 (57,6%)	15 (46,9%)	0.388
- Acute liver failure	11 (16,9%)	5 (1522%)	6 (18.8%)	0.699
- Pneumothorax	1 (1,5%)	0 (0%)	1 (3%)	0.306
- Ischemic gut	1 (1,5%)	0 (0%)	1 (3%)	0.306
- Sternal wound abscess	1 (1,5%)	0 (0%)	1 (3%)	0.306
- HIT	19 (29,2%)	13 (39.4%)	6 (18.8%)	0.067
- Acute abdomen	3 (4.6%)	0 (0%)	3 (9.8%)	0.072
- Pneumonia	26 (40%)	15 (45.5%)	11 (34.8%)	0.362
- AV Block III	4 (6,2%)	1 (3.0%)	3 (9.8%)	0.287
- Heart rupture	3 (4,6%)	1 (3.0%)	2 (6.3%)	0.536
- RV failure	5 (7,7%)	3 (9.1%)	2 (6.3%)	0.667
Therapies during ECMO support				
Packed red blood cells transfusion (units)	17.0±10.5	18.2±10.3	15.8±10.7	0.366
Fresh frozen plasma transfusion (units)	16.6±10.6	18.2±10.2	13.8±10.8	0.103
Platelet transfusion (units)	5.0 (2.0-9.0)	8.0 (3.0-10.0)	4 (2.0-6.8)	0.028
Argatroban	17 (26.2%)	9 (27.3%)	8 (25.0%)	0.835
Levosimendan	17 (26,2%)	7 (21.2%)	10 (31.3%)	0.357
Epinephrin	60 (92,3%)	33 (100%)	27 (84.4%)	0.018
Enoximone	40 (61,5%)	25 (75.8%)	15 (46.9%)	0.017
Dobutamin	12 (18,5%)	2 (6.1%)	10 (31.3%)	0.009
Intra-aortic balloon pump	3 (4.6%)	1 (3.0%)	2 (6.3%)	0.536
Impella CP device	7 (10.8%)	0 (0%)	7 (21.9%)	0.004
ECMO settings				
ECMO flow (l/min)	3.9±0.9	3.8±0.8	4.0±1.0	0.242
Rotational speed (r/min)	3519±562	3389±537	3657±564	0.056
Gas flow (l/min)	3.5 (2.5-4.0)	3.5 (2.5-4.0)	3.0 (2.0-5.0)	0.876
Oxygen fraction in the ECMO device (%)	60 (50-70)	60 (50-60)	70 (55-80)	0.032

ECMO, extracorporeal membrane oxygenation; ICU, intensive care unit; LOS, length of stay; HIT, heparin-induced thrombocytopenia; AV, atrio-ventricular; RV, right ventricle

The rates of most complications, such as stroke, acute kidney injury, liver failure, abdominal compartment syndrome, pneumothorax, acute abdomen, pneumonia, AV-blockage, rupture of the left ventricle, right ventricular failure etc. were comparable between the two groups. However, we detected significant differences in bleeding events (84.8% vs. 56.3%, *p* = 0.011), which occurred more often in the cECMO group, and leg ischemia (3.0% vs. 18.8%, *p* = 0.041) as the most recurring problem in the pECMO group (Table 3).

When bleeding is considered as a separate problem, hemothorax (53.1% vs. 21.9%, *p* = 0.013) and mucosal/nasal bleeding (48.5% vs. 12.5%, *p* = 0.002) were particularly prevalent in the cECMO group. Furthermore, patients in the cECMO group were more likely to undergo a re-thoracotomy (1.0 (0–1.5.5) vs.0 (0–0.8.8), *p* = 0.001). Consequently, a higher incidence of bleeding events was observed in the cECMO group, resulting in an increased need for platelet transfusions (8.0 [3.0–10.0] vs. 4.0 [2.0–6.8.0.8], *p* = 0.028). The utilisation of preoperative anticoagulant medication (aspirin, clopidogrel/ticagrelor, direct-acting oral anticoagulants [DOAC], and marcoumar) was observed to be equivalent in both cohorts, and could not impact the observed bleeding differences (Table 1).

With regard to the ECMO device values, the observed flow rate (3.8 ± 0.8 versus 4.0 ± 1.0 l/min, *p* = 0.242) remained consistent (Table 3). However, the rotational velocity (r/min) exhibited a marginal increase in the pECMO cohort (3389 ± 537 vs. 3657 ± 564, *p* = 0.056). The gas flow (l/min) exhibited no significant difference between the two groups (3.5 (2.5–4.0.5.0) vs. 3.0 (2.0–5.0), *p* = 0.876). However, patients in the pECMO group required a higher oxygen fraction in the ECMO device (60 (50–60) % vs. 70 (55–80) %, *p* = 0.032).

Analysing the laboratory values on day 3 after ECMO initiation, we found higher CRP levels (166.1 [100.7–213.4.7.4] vs. 91.5 [67.3–140.7.3.7], *p* = 0. 014) in cECMO patients, while pECMO patients exhibited higher creatine kinase (CK) levels (9.4 [5.0–28.9.0.9] vs. 40.0 [5.1–68.1], *p* = 0.161) (Table 4).

**Table 4:** Laboratory parameters during ECMO support of the entire population and for the cECMO and pECMO groups

Laboratory parameters after 3 days of the ECMO support				
Parameters	**Overall** *n* = 42	**cECMO group**, *n* = 22	**pECMO group**, *n* = 20	**p-value**
pH	7.40 (7.40–7.50)	7.40 (7.40–7.50)	7.44 (7.40–7.50)	0.751
Lactate	1.6 ± 0.8	1.4 ± 0.5	1.8 ± 1.0	0.101
Hemoglobin (mmol/l)	6.0 ± 0.5	6.0 ± 0.4	6.0 ± 0.6	0.977
Leucocyte (10^9^/L)	13.6 (10.0–16.0)	13.4 (9.6–15.3)	13.9 (10.3–19.9)	0.442
Platelet count (10^9^/L)	102.0 ± 32.7	100.4 ± 34.1	104.1 ± 31.7	0.724
CRP (mg/L)	119.1 (77.9–210.2.9.2)	166.1 (100.7–213.4.7.4)	91.5 (67.3–140.1.3.1)	0.014
CK (µmol/(s•L)	16.0 (5.0–46.9.0.9)	9.4 (5.0–28.9.0.9)	40.0 (5.1–68.1)	0.161
CK-MB (µmol/(s•L)	0.6 (0.3–1.2)	0.5 (0.2–0.8)	0.7 (0.4–1.4)	0.116
CK-MB (%)	2.9 (1.5–5.7)	3.1 (1.5–5.9)	2.1 (1.4–5.4)	0.486
PTT (s)	47.2 ± 9.4	46.6 ± 8.9	47.9 ± 10.2	0.679
INR	1.2 (1.1–1.3)	1.2 (1.1–1.2)	1.2 (1.1–1.4)	0.184
Antithrombin	76.8 ± 15.5	73.7 ± 17.4	80.2 ± 17.8	0.198
D-Dimer	3.0 (1.3–10.9)	4.4 (1.4–15.5)	2.4 (1.4–15.8)	0.607
Fibrinogen (g/L)	4.8 ± 1.5	4.8 ± 1.0	4.8 ± 1.8	0.959
LDH (µmol/ls)	12.3 (8.8–16.1)	13.0 (6.8–17.0)	11.8 (10.3–14.5)	0.849
ALT (µmol/ls)	1.5 (0.8–9.1)	1.2 (0.7–6.2)	2.2 (1.0–15.1.0.1)	0.241
Creatinine (µmol/l)	138.5 (72.8–210.5.8.5)	138.5 (72.8–244.8.8.8)	130.0 (71.0–170.7.0.7)	0.870
Total bilirubin (µmol/l)	18.0 (12.2–40.1)	19.0 (11.9–42.9)	15.6 (12.2–39.5)	0.821

### Univariate and multivariate logistic regressions

The construction of a predictive model was facilitated by the implementation of a logistic regression analysis. Preliminary univariate analysis results indicated that known hypertension prior to myocardial infarction (odds ratio [OR]: 5.000; 95% confidence interval [CI]: 1.366–18.300, *p* = 0.015), the presence of heparin-induced thrombozytopenia (HIT) during ECMO support (OR: 0.282; 95% CI: 0.092–0.863; *p* = 0.027), pECMO (OR: 0.2326; 95% CI: 0.116–0.914; *p* = 0.033), and high lactate level (mmol/l) on day 3 of ECMO (OR: 0.283; 95% CI: 0.085–0.940, *p* = 0.039) were identified as factors associated with 60-day mortality in adult patients receiving ECMO for severe CS.

Multivariate analysis was subsequently performed to create a mortality prediction model using variables that were significant (*p* ≤ 0.05) or tending to be significant (*p* ≤ 0.1). It was not possible to include lactate values on day 3 in the analysis, because 23 patients had already died by this time and would be therefore excluded during this analysis. Nevertheless, according to univariate logistic regression analysis, no significant effect on mortality was observed in this study when considering lactate levels prior to ECMO. According to multivariate analysis, the presence of arterial hypertension prior to myocardial infarction (OR: 14.815; 95% CI: 2.250–97.547.250.547; *p* = 0.005), pECMO (OR: 0.150; 95% CI: 0.030–0.755, *p* = 0.021), and the most severely impaired LVEF (OR: 1.080; 95% CI: 1.002–1.166, *p* = 0.046) were considered significant factors for mortality (Table 5).

**Table 5:** Total population regression analysis by 60-day survival

Variables in the Equation								
	B	S.E.	Wald	df	Sig.	CI	95% C.I.for EXP(B)	
							Lower	Upper
Age	−0.079	0.042	3.583	1	0.058	0.924	0.852	1.003
Peripheral ECMO	−1.807	0.825	5.293	1	0.021	0.150	0.030	0.755
Hypertension prior to AMI	2.696	0.962	7.858	1	0.005	14.815	2.250	97.543
Leg ischemia	2.645	1.451	3.324	1	0.068	14.085	0.820	241.968
AKI with dialysis	1.318	0.722	3.332	1	0.068	3.736	0.907	15.378
HIT	−0.627	0.782	0.642	1	0.423	0.534	0.115	2.476
LVEF (%)	0.077	0.039	3.995	1	0.046	1.080	1.002	1.166
Constant	0.517	2.826	0.033	1	0.855	1.677		

ECMO, extracorporeal membrane oxygenation; AKI, acute kidney injury, HIT, heparin-induced thrombocytopenia; LVEF, left ventricular ejection fraction; AMI, acute myocardial infarction

The mortality prediction model explained 51.0% (Nagelkerke R2) of in-hospital mortality variance, correctly classifying 81.5% of the cases (sensitivity: 86.8%; specificity: 74.1%). The Hosmer-Lemeshow test assessed the goodness of fit and indicated a good model fit (χ ² (7) = 8.181, *p* = 0.317).

## Discussion

In this retrospective single-center study of 65 patients, we compared different cannulation strategies for ECMO support in patients with infarct-related cardiogenic shock. The findings of our study indicated that the 60-day mortality rate was significantly lower in patients who had been cannulated via the end-to-side ascending aorta prosthetic graft for cECMO compared to those who had undergone pECMO (45.5% vs. 71.9%, *p* = 0.031). Concurrently, central cannulation with this technique was observed to be associated with a higher incidence of bleeding (84.8% vs. 56.3%), predominantly in the form of hemothorax (53.1% vs. 21.9%), which consequently resulted in an increased need for blood products. In our experience, bleeding was frequently observed at the anastamois between the aorta and the ECMO prosthesis, particularly in the early stages of performing the new technique, which could be related to the learning curve. The results improved with more meticulous hemostasis and utilisation of a specialized adhesive, as well as platelet transfusions. The high prevalence of leg ischaemia in the pECMO group (18.8%) can be attributed to either the presence of underdiagnosed peripheral arterial disease involving stenosis of the lower limb arteries in patients admitted with AMI, or to the complexity of femoral arterial cannulation, as well as the concomitant application of the Impella CP device with ECMO on the femoral arteries. It is important to note at the outset the disparity of the groups in terms of revascularisation methods, with a significant proportion of patients in the pECMO group having undergone PCI prior to va-ECMO implementation. This could be justified as an early reavasuclarisation was favoured using PCI whenever possible. A substantial number of studies have demonstrated that treating of multivessel coronary heart disease with CABG is associated with reduced hospital mortality, decreased rates of readmission to hospital, reduced coronary reinterventions, and enhanced long-term survival [13, 14]. Concurrently, a recent meta-analysis has revealed that, among patients afflicted with left main coronary artery disease, there was no statistically significant discrepancy in one-year or five-year all-cause mortality between PCI and CABG [15]. Regression analysis in the present study revealed that the method of revascularisation had no significant impact on 60-day mortality rates (OR: 1.571; 95% CI: 0.469–5.270, *p* = 0.464).

In a meta-analysis done by Raffa et al., including 1691 patients, comparing central cannulation (right atrial to ascending aorta) versus peripheral cannulation in patients with postcardiotomy CS und non-postcardiotomy CS there was no difference in the analysis between the two techniques regarding all-cause mortality. On the other hand, the central ECMO was associated with more risk of bleeding, continuous venovenous hemofiltration, and increased need for blood products [16]. The 17 selected studies included in the meta-analysis provided data for the analysis of in-hospital all-cause mortality, which accounted to 64.2% in the overall analysis within the specified time frame [16]. It is noteworthy to mention that the study included only 96 patients (5.7%) with infarct-related CS. Nevertheless, the findings of the study indicated an increased incidence of bleeding and a greater requirement for blood products in patients undergoing cECMO confirming our results.

In the single-center study conducted by Djordjevic et al., central (right atrial to ascending aorta) and peripheral ECMO were compared in patients with postcardiotomy CS, including 31% of patients with an urgent CABG procedure. The 30-day mortality rate was comparable between the two cohorts, with nearly 70% mortality in both [8]. The findings of this study revealed that on-site complications associated with ECMO were significantly more prevalent in patients who underwent cECMO. These complications, primarily bleeding events, were observed to be more prevalent in this patient cohort, frequently necessitating reoperations. Subsequently, the administration of fresh frozen plasma was also more prevalent in the cohort with cECMO.

The previously mentioned central cannulation with prosthetic graft as Y-ECMO was described in patients with critical failure of other organs (such as 2nd or 3rd degree pulmonary oedema and/or stage 2 or 3 of AKI) who had already undergone pECMO for salvage. Central Y-ECMO was performed for re-cannulation to enhance organ perfusion in six patients (three patients with infarct-related CS and three patients with myocarditis). This intervention resulted in an improvement in the patients’ condition overall, with an intrahospital mortality of 16.7% (one patient) [11].

Cannulation of the femoral artery is occasionally unfeasible due to severe peripheral arterial disease. Consequently, efforts have been made to establish alternative methods of ECMO cannulation. A recent study by Vale et al. presents data on subclavian artery cannulation, whereby arterial blood can be returned via an end-to-side graft into the right subclavian or axillary artery [17]. The former demonstrated that the 90-day mortality rate was comparable between the two groups (54% vs. 58%). Cannulation of the axillary artery for ECMO has been shown to be associated with a significantly lower incidence of local infections, limb and bowel ischaemia, and pulmonary edema episodes. However, this method of cannulation has been demonstrated to carry an increased risk of stroke when compared to femoral artery cannulation [17]. In accordance with the findings of numerous studies in this field, the present analysis revealed that only 14% (46 patients) in this study presented with AMI.

Pisani et al.. reported a 30-day relatively high survival rate of 63.8% in patients with axillary cannulation for ECMO [18]. These results also demonstrated that implantation following coronary artery bypass grafting was associated with poor outcomes. Consequently, the high survival rate observed in this study may be attributed to the selection bias of the study population, as the majority of patients, who received circulatory support for cardiogenic shock due to cardiac arrest, were excluded from the analysis, having undergone urgent femoral cannulation.

A further disadvantage of pECMO is that blood moves in the opposite direction to normal physiological circulation; retrograde from the femoral or iliac artery back to the thoracic aorta and supraoartic vessels in addition to the blood pumped from the heart. As a result, in patients receiving pECMO, a watershed region is present, defined as the area in the aorta where the two blood flows converge. The precise location of this watershed region varies depending on the relative performance of left ventricle (LV) in relation to ECMO flow, extending from the aortic root to the diaphragm [7, 19]. Consequently, when the watershed is situated distal to the left subclavian artery, there is a potential risk of profound hypoxemia of the brain, heart and upper extremities. In extreme cases, this may result in the development of Harlequin Syndrome, otherwise known as North-South Syndrome. In this condition, blood with low oxygen levels from the lungs (e.g. due to pulmonary oedema, infection, internal disease, etc.) is pumped to the upper body and brain by the LV [20].

One potential drawback of veno-arterial ECMO support is the increase in afterload dependent on unphysiological retrograde arterial flow, which may result in LV distension and subsequent pulmonary oedema [21]. A variety of percutaneous and surgical treatment options, in combination with drug therapy, have been described as a viable approach for the prevention and treatment of pulmonary oedema, with a favourable impact on patient outcomes [7, 22].

The primary pharmacological intervention for left ventricular unloading is inotropic agents. The adverse effects of such therapy, manifesting as increased oxygen consumption and exacerbation of ischaemia, have been extensively reported [23]. Nevertheless, observations have demonstrated that low-dose inotrope therapy during ECMO support results in enhanced aortic valve opening, thereby contributing to left ventricular unloading [7]. In the present study, all patients in the cECMO group received epinephrine, whereas in the pECMO group it was used to a lesser extent and was replaced by dobutamine (Table 1). Epinephrine exhibited a similar effect on cardiac index to the combination of dobutamine and norepinephrine, however, it was more likely to induce tachycardia and lactate acidosis [24]. Nonetheless, the dosage of inotropes was low during ECMO support and increased over a few hours before ECMO weaning. Concomitant with the administration of conventional inotropes, the utilisation of enoximone was observed to be more prevalent during weaning and immediately following explantation from support in the cECMO group. In addition, the other inotropic agent, levosimendan, was utilised for the weaning process from ECMO to an equivalent extent in both groups.

The utilisation of an intra-aortic balloon pump (IABP), which has the capacity to facilitate left ventricular unloading, was observed in three patients across both cohorts. This therapeutic approach failed to demonstrate a survival benefit in patients undergoing ECMO [25].

The utilisation of a percutaneous transaortic ventricular assist device (Impella CP) inserted via the femoral artery was exclusively observed within the pECMO cohort (7 cases [21.9%] vs. 0 cases [0%], *p* = 0.004). This occurred frequently before the initiation of ECMO for the stabilisation of patients during PCI, and the device was then explanted within a period of 1–3 days. As demonstrated by Koeckert et al., this approach has been effective in reducing the LV end-diastolic diameter and pulmonary oedema [26]. The primary objective of this therapeutic intervention is to reduce pressure in the LV and the pulmonary veins, while concomitantly providing antegrade flow support to the aortic root. The utilisation of the Impella device has been demonstrated to facilitate early termination of ECMO, assuming adequate oxygenation, resulting in further unloading of the LV and pulmonary veins. The data presented in several reports on combined support with peripheral ECMO and Impella demonstrated enhanced survival in comparison with peripheral ECMO alone [27, 28]. A recent systematic review and meta-analysis of the use of ECMO combined with the Impella device for cardiogenic shock following acute myocardial infarction showed a survival benefit compared with ECMO-IABP, but patients were more likely to have bleeding complications, vascular complications, the need for new renal replacement therapy, and intracranial hemorrhage [29].

Nevertheless, the findings of our study demonstrated that, despite the utilisation of the Impella CP in a small number of patients in the pECMO group (21.9%), the survival rate remained lower. This is constrained by the relatively limited sample size of patients with Impella devices; therefore, it is not possible to ascertain the relative merits or drawbacks of the Impella CP system.

Our study’s limitations are its retrospective nature, the modest number of patients, and the heterogeneity of the groups with regard to left ventricular unloading. Notwithstanding these limitations, the study features a distinctive cohort of patients with infarct-related CS, a subject that has received little attention in the present literature. The present study has demonstrated that cECMO may be a useful procedure in situations where weaning from the heart-lung machine is not possible during urgent CABG. The central aortic cannulation technique we use with a prosthesis and femoral venous cannulation also facilitate in early recovery in comparison to cECMO with direct cannulation (atrial to aorta) also not requiring a reentry surgery to remove the cannulas. The hypothesis that the utilisation of cECMO results in enhanced hemodynamics and improved upper body oxygenation has yet to be substantiated by further prospective research.

## Conclusion

The choice of a prosthetic graft for aortal cannulation in cECMO patients with infarct-related CS demonstrated a significantly lower 60-day mortality compared with pECMO, despite showing a significantly higher incidence of major bleeding, and may be considered as a reliable method of MCS.

## Data Availability

The data that underpin the results presented are available by contacting the corresponding author (boris.kuzmin@med.ovgu.de). Due to privacy and ethical restrictions, the data are not publicly available.

## Abbreviations

AKI: Acute kidney injury
AMI: Acute myocardial infarction
BMI: Body mass index
CRRT: Continuous renal replacement therapy
CABG: Coronary artery bypass graft
cECMO: Central extracorporeal membrane oxygenation
CI: Confidence interval
CKD: Chronic kidney disease
DOAC: Direct-acting oral anticoagulants
ELSC: Extracorporeal life support
ECMO: Extracorporeal membrane oxygenation
HIT: Heparin-induced thrombozytopenia
ICU: Intensive care unit
INR: International normalized ratio
LVAD: Left ventricular assist device
LVEF: Left ventricular ejection fraction
MCS: Mechanical circulatory support
PCI: Percutaneous coronary intervention
pECMO: Peripheral extracorporeal membrane oxygenation
aPTT: Activated partial thromboplastin time
RRT: Renal replacement therapy
